# Assessing Social Cognition of Persons with Schizophrenia in a Chinese Population: A Pilot Study

**DOI:** 10.3389/fpsyt.2017.00302

**Published:** 2018-01-10

**Authors:** Panmi M. T. Lo, Andrew M. H. Siu

**Affiliations:** ^1^Occupational Therapy Department, Castle Peak Hospital, Hospital Authority, Hong Kong, Hong Kong; ^2^Department of Rehabilitation Sciences, The Hong Kong Polytechnic University, Hong Kong, Hong Kong

**Keywords:** assessment, social cognition, schizophrenia, validation, Chinese

## Abstract

Social cognition is a core limiting factor of functional recovery among persons with schizophrenia. However, there is a lack of standardized and culturally relevant assessment tools for evaluating social cognitive performance in Chinese persons with schizophrenia. The purposes of this study were to (1) develop and validate two social cognitive instruments, the Chinese Facial Emotion Identification Test (C-FEIT) and the Chinese Social Cognition and Screening Questionnaire (C-SCSQ), that assess three key domains of social cognition and (2) to evaluate preliminary psychometric properties of the two assessments. The results demonstrated that the C-FEIT and the social cognitive subscales of C-SCSQ possess satisfactory content-related validity and test–retest reliability (ICC ranging from 0.76 to 0.85). Subscales of the C-FEIT and the C-SCSQ showed low to medium correlation with two concurrent neurocognitive measures (absolute values of r ranging from 0.22 to 0.45) and concurrent measures of functional performance (absolute values of r ranging from 0.22 to 0.46). Our findings generally support the use of the C-FEIT and the C-SCSQ as reliable and valid tools for assessing emotion perception, theory of mind (intention-inferencing), and hostile attributional style, which are the key outcome indicators of social cognitive interventions for persons with schizophrenia.

## Introduction

Schizophrenia is a severe psychiatric illness characterized by marked deficits in a wide array of functional areas. A key focus of current research is to identify factors limiting function in schizophrenia. In the past decade, there has been increasing research interest in examining social cognition as a predictor of functional outcomes in rehabilitation. There is growing evidence that social cognition mediates the relationship between neurocognition and functional outcomes, and it is possible that improving social cognitive deficit or reducing social cognitive bias could make a positive impact on social and work adjustment of persons with schizophrenia (1–6).

Social cognition refers to how people think about themselves and others in the social world (7). It is conceptualized to encompass a number of cognitive processes that underlie social interactions, including perceiving, interpreting, and responding to the intentions, dispositions, and behaviors of others (8). These social cognitive processes had been further examined and confirmed in social neurosciences study, which revealed the underlying involvement of different but related brain structures (9). While, in the past, there has been controversy over what social cognitive processes should cover, social cognition is now generally defined as encompassing four key domains: emotion perception (EP)/emotion processing, theory of mind /mental state attribution, attributional style, and social perception (10, 11).

This study aims to translate, adapt, and validate a set of two instruments, the Facial Emotion Identification Test (FEIT) and the Social Cognition and Screening Questionnaire (SCSQ), for assessing social cognition in persons with schizophrenia. This study addresses several issues in social cognitive assessment with Chinese populations. First, there are uncertainties on the exact construct measured in some of the existing social cognitive assessments. For instance, the Reading the Mind in the Eyes Test (12), which intended to measure ToM ability, appeared to measure emotion recognition ability or empathy more than higher level mental state attribution (13). The Mayer–Salovey–Caruso Emotional Intelligence Test, which was originally designed to assess emotion intelligence ability (14), required participants to rate effectiveness of emotion management strategies in social scenarios. This task though requires certain level of ability to perceive emotions; it, however, did not assess facial EP directly with the use of photos. There are also questions on whether the Attributional Style Questionnaire (15) assessed the specific attributional style (16) observed among persons with schizophrenia. Based on this review, we selected the FEIT and SCSQ in this validation, as the two tests are able to cover most domains of social cognition relevant to the assessment of persons with schizophrenia. The FEIT covers the assessment of EP, while the SCSQ assesses theory of mind and attributional style, as well as a closely related construct, jump-to-conclusion that are commonly observed in persons with schizophrenia (5, 17, 18).

Second, there are very few Chinese-language version of social cognitive assessments. Many studies of social cognition with Chinese populations focused on EP (19–21), but did not cover assessment of other aspects of social cognition. As it is well documented that culture impacts on development of different social cognitive domains and possibly impacts the related social cognitive performance (22–25), this study will review the cultural relevance of common social cognitive assessments before the assessments are translated for application with Chinese populations.

Third, we found that several adaptations of the FEIT are needed to prevent bias in testing EP. A comprehensive photo set should include six basic emotions faces (26, 27) and neutral facial expression faces (28). The photo set should include an equal number of posers of both genders to display emotions as the use of a photo set with single gender poser could lead to bias in assessment result (29, 30). In addition, the posers should come from both Western and Asian cultures, in order to prevent cultural biases in assessment. In this study, we would make changes to the test photo set in FEIT to prevent these biases in measurement of EP.

In summary, we observed a number of limitations in current instrument for assessing social cognition in Chinese persons with schizophrenia. We propose the combined use of SCSQ and FEIT to form a set of social cognitive assessments that could be administered to target clients within a reasonable timeframe. The specific objectives of this study are to (1) translate and validate two social cognitive instruments, the Chinese version of the Facial Emotion Identification Test (C-FEIT) and the Chinese Social Cognition and Screening Questionnaire (C-SCSQ) to assess the three key domains of social cognition and (2) examine the preliminary psychometric properties of the two instruments, including reviewing the content validity and cultural relevance for C-SCSQ, and reviewing the reliability, concurrent validity and known-groups validity of both instruments.

## Materials and Methods

### Participants

Two groups of participants were recruited for the study, including patients with schizophrenia and non-psychiatric control. For the patient group (*n* = 62), the inclusion criteria were (1) aged 18–60 years old and (2) diagnosis of schizophrenia according to the International Classification of Diseases version 10 (ICD-10). The exclusion criteria were (1) dual diagnosis of schizophrenia, such as a neurological disorder, a developmental disability, or a substance abuse problem; (2) diagnosis of intellectual disabilities; and (3) admission to in-patient psychiatric treatment or a change in psychiatric medication within the last 30 days. The inclusion and exclusion criteria of the patient group were confirmed by retrieving information from hospital medical record. Diagnosis of schizophrenia was made by psychiatrist using ICD-10.

For the known-groups method of validation, we planned to conduct a comparison of social cognition of persons with schizophrenia and non-psychiatric controls. A total of 20 non-psychiatric control participants were planned. This sample size is calculated by PASS 12 (31) with a power of 0.80 and effect size set at 0.90. The effect size of 0.90 is set based on effect sizes of group differences between patients and controls in EP, ToM, and AS in previous studies (ranged from 0.90 to 2.47) (16, 32, 33). A group of non-psychiatric control participants (*n* = 19), matched on age and gender characteristics with some of the participants with schizophrenia, were recruited from a local community center. Only those with no history of psychiatric illness were recruited as non-psychiatric controls. The inclusion and exclusion criteria of the non-psychiatric control participants were determined by semi-structured interviews.

### Procedures

Ethical approval to conduct the study was obtained from the Departmental Research Committee of the Department of Rehabilitation Sciences, the Hong Kong Polytechnic University and the Cluster Clinical Research Ethics Committee, New Territories West Cluster of the Hospital Authority. Written informed consent to participate in our study was obtained from participants before the start of data collection. A team member (a researcher or an assistant) briefed the potential participants about the purpose and procedures of the study, then obtained the written informed consent. We administered all social cognitive, neurocognitive, symptoms, and functional measures to the patient group. To allow us to collect data for examining the test–retest reliability, a subgroup of the patient group (*n* = 17) completed the two social cognitive instruments (C-FEIT and C-SCSQ) twice with a 1-week interval in between. For the non-psychiatric control group (*n* = 19), we administered only the social cognitive tasks (C-FEIT and C-SCSQ), to investigate the known-groups validity of the two instruments.

### Instruments

We collected basic background information (age, education) of the participants through brief interviews or by extracting data from case records. The two social cognitive assessment tasks adopted in this study are the FEIT and the Social Cognition Screening Questionnaire (SCSQ).

#### Facial Emotion Identification Test

The FEIT is widely used to assess facial EP ability (34). The set of FEIT test photos used in current study consists of 21 photos: 12 photos showing the six basic emotions (happiness, sadness, anger, disgust, fear, and surprise) and nine photos conveying neutral emotions (used as controls). The 12 basic-emotion photos were selected from the Japanese and Caucasian Facial Expression of Emotion (JACFEE) photo set (35), which demonstrated acceptable levels of agreement in a study with Chinese subjects (36). The nine neutral-emotion photos were selected from the Japanese and Caucasian Neutral Faces (JACNeuF) collection (35, 37).

In constructing the C-FEIT used in this study, we made a few changes to the original FEIT to address some methodological issues identified in previous studies. First, we used photos displaying disgust to replace shame in the FEIT, as disgust is widely regarded as one of six basic emotions (27), and disgust is more commonly use in EP studies of persons with schizophrenia than shame (30). Second, we used an equal number of photos displaying different emotions. Third, we used photo sets from both the JACFEE and JACNeuF (photos with neutral emotion), this photo set is one of the gold standards of facial expressions (35). In fact, a similar photo set had been validated for use with Chinese population, and agreement level was acceptable (*N* = 120) (36). It would be best to use photos of Chinese faces to portray the six basic emotions in the test, but there is not yet any validated photo set of Chinese faces that consisted of six basic emotions (38).

A set of Chinese-language instructions was used to guide the standard administration of the test. The photo sets were presented in random order on a notebook computer or a desktop computer. Several parameters were standardized during administration. First, the time of exposure and time of rest in between two photos were each fixed at 10 s, in accordance with the standards used in similar studies. Second, the photo sets were presented on either a desktop computer or a notebook (using Microsoft PowerPoint) with a screen size of less than 18 inches, and with an arm’s length between the participant and the screen. These settings were used to approximate a face size of the subject pictured in the photo to that of a face as it would appear in day-to-day conversation. After viewing each photo, the participant was required to select which of the seven emotions (six basic emotions or the neutral option) was conveyed in the photo, and to record the answer on the answer sheet. The participants’ responses were recorded and marked as correct or incorrect.

#### Social Cognition and Screening Questionnaire (SCSQ)

The SCSQ is designed to assess three key aspects of social cognition: ToM, jumping-to-conclusions (JTC) bias, and paranoid attributional style. The instrument screens for neurocognitive deficits and the patient’s needs for social cognitive intervention (39). The SCSQ presents interpersonal vignettes that describe ambiguous interpersonal situations. The examiner presents the vignettes to the participant orally within 10 s, then the participant is required to answer three yes/no questions. Two of the yes/no questions test the participant’s ability to recall details in each vignette, and the total number of correct answers is summed as the “neurocognitive score” (NC). The score range is 0–20. The remaining yes/no questions involve an intention-inferencing task that tests the participant’s ability to infer a character’s intentions from information in the vignette. The total number of correct answers is summarized as the “perspective-taking score” or “theory of mind” (ToM). The score range is 0–10. The confidence-judgment question assesses the participant’s tendency to make overconfident judgments, which contributes to the “JTC score” (JTC). The “paranoid/hostile attributional bias score” (PAS) is calculated by summing the incorrect perceptions in vignettes 2, 3, 5, 6, and 9 (incorrect perceptions are those answers with a score range of 0–5).

With approval from the original author, we hired a qualified translator to translate the original English version of the SCSQ into Chinese using the idiomatic translation method. A four-member expert panel was set up to (1) appraise the quality of the translation, evaluate the semantic equivalence of the two versions, examine the fluency and clarity of the translation, and provide suggestions to improve the translated instrument and (2) evaluate the content-related validity (relevance and representativeness) of the C-SCSQ. The panel members included mental health professionals with at least 10 years of clinical experience, including psychiatrists, clinical psychologists, and occupational therapists.

#### Psychiatric Symptoms

The mental status of the patients was assessed using the Brief Psychiatric Rating Scale, Expanded Version (BPRS-E) (40). The BPRS-E generates both a total score and a paranoia score. The Paranoia scale (41) consists of five items assessing hostility, suspiciousness, tension, uncooperativeness, and excitement. The paranoia score is used for studying concurrent validity with the paranoid/hostile attributional style (PAS) of the SCSQ. The BPRS-E possessed satisfactory inter-rater reliability of 0.72(42).

#### Neurocognitive Measures

We selected several subtests of the MATRICS Consensus Cognitive Battery (43) for studying concurrent validity with the Neurocognitive (NC) scale of the SCSQ. The MATRICS Consensus Cognitive Battery measures speed of processing, verbal memory, and verbal fluency (VF). The speed of processing was assessed using the Trail Making Test Part A, and VF was measured by a categorical fluency test using animal naming (44). Verbal learning was measured by the Hopkins Verbal Learning Test, Revised Version (HVLT-R) (45).

#### Functional Measure

The Chinese Work Personality Profile (CWPP) is a situational assessment tool for assessing work maintenance behavior in a simulated or actual work setting. It consists of 58 items and it has five subscale scores: task orientation, social skills, self-control, attitude, and personal appearance. The CWPP is used as a concurrent measure of function and it is expected to correlate with social cognitive measures. The CWPP has demonstrated good discriminant and predictive validity in previous studies (46), and it has been found to be closely linked to social cognitive measures (6).

### Statistical Analysis

The test–retest reliability of the C-FEIT and the subscales of the C-SCSQ were examined using the Intraclass Correlation Coefficient (ICC), using a two-way mixed model of ICC for evaluating consistency (47). The internal consistency of C-FEIT and C-SCSQ were examined using Cronbach’s alpha. For known-groups validity, the *t*-test was used to compare the differences in social cognitive performances between the patient group and the non-psychiatric control group. Correlation coefficients were used to investigate the concurrent validity with neurocognitive and clinical variables, and the concurrent validity with functional performance.

## Results

Table 1 presents the demographic and clinical characteristics of the patient sample. Around half (45.2%) of the participants were males. They had an average duration of illness of 12.04 years. The majority (90.3%) of subjects received atypical medication, and we did not include further information on chlorpromazine-equivalent dosage because the effect of antipsychotic medication on cognitive and social cognitive disorders remains uncertain (48). The BRPS scores showed that the participants had few symptoms and were largely mentally stable. The social cognitive abilities, neurocognitive, and indexes of functional performance are also presented in Table 1.

**Table 1:** Demographic and clinical characteristics (*N* = 62).

Variables	%	M (SD)
**Demographics**		
Gender (male)	45.2	
Medication (atypical)	90.3	
Age		37.97 (11.8)
Years of education		10.67 (2.8)
Age of onset		25.93 (9.4)
Duration of illness		12.04 (9.3)

**Clinical Symptoms**		
BPRS total		1.24 (0.2)
BPRS paranoia scale		1.16 (0.3)

**Social Cognitive Measures**		
Chinese Facial Emotion Identification Test (FEIT)		14.50 (3.9)
NC		14.11 (3.2)
Theory of mind (ToM)		6.32 (1.8)
JTC		2.66 (0.8)
Paranoid/hostile attributional style (PAS)		1.68 (1.1)

**Neurocognitive Measures**		
TMT^a^		57.97 (23.8)
HVLT^b^		19.92 (6.53)
Verbal Fluency^b^		17.60 (5.2)

**Chinese Work Personality Profile**		
Task orientation		74.37 (16.11)
Social skills		44.13 (9.80)
Self-control		22.03 (4.3)
Acceptance of supervision		27.27 (5.9)
Personal presentation		6.80 (1.2)

*FEIT, emotion perception of FEIT, ToM: Theory of mind, JTC: jumping-to-conclusions, PAS: paranoid/hostile attributional style, NC, neurocognitive subscale of SCSQ, TMT, Trail Making Test A, HVLT, Hopkins Verbal Learning Test, VF, Verbal Fluency, BPRS total: total score of Brief Psychiatric Rating Scale, BPRS Paranoia scale: item 6 (tension), item 10 (hostility), item 11 (suspiciousness), item 14 (uncooperativeness), and item 17 (excitement) of BPRS*.

### Content Validity and Cultural Relevance of the C-SCSQ

The content validity and cultural relevance of the C-SCSQ were reviewed by an expert panel. All panel members were bilingual (Chinese and English) and had at least 10 years of experience in mental health practice. All the experts agreed that the Chinese translation of the SCSQ was semantically equivalent to the original English version, and most of them (75%) agreed that the Chinese translation was fluent. All agreed that the translated version was clear in presentation. Several modifications were suggested by the experts to address issues of cultural relevance. These included replacing “spaghetti” with “fried rice” in vignette 3; replacing “Bingo Game” with “Buy Mark Six (name of local lottery),” and “Susan/Stan” with “Mei Ling/Wai Man” (common Chinese names) in vignette 8; and adjusting the price of toothpaste mentioned in vignette 6 from US dollars to Hong Kong dollars. Most (75%) experts agreed that the translated SCSQ was culturally relevant with the above modifications.

The experts rated most (over 90%) items as having satisfactory content relevance, which exceeded the standard of 75% agreement. The only exception was item 9A, which only 50% of the panel members agreed was relevant. As for content representativeness, all the experts agreed that the theory-of-mind and JTC subscales were representative of social cognition, while 75% of the experts agreed that the neurocognitive and paranoid attributional bias subscales were representative. Based on the results of the content-related validity, three items (9A, 9C, and 2B) and three phrases in vignettes 5, 9, and 10 were modified according to the suggestions of the experts before the C-SCSQ was finalized and administered to the study samples.

### Reliability

Test–retest reliability was investigated by administration of the two social cognitive tasks twice to 17 patients with a 1-week interval between tests. The test-retest reliability scores—as indicated by Intraclass Correlation Coefficients of the subscales—were all above the standard of 0.75, except the neurocognitive subscale (ICC = 0.67) (Table 2) (49). For internal consistency, the Cronbach’s alpha for C-FEIT was 0.776 and for C-SCSQ total score including NC, ToM, and AS subscales was 0.630.

**Table 2:** Descriptive statistics and test–retest reliability of C-FEIT and C-SCSQ (*N* = 17).

Subscales	First administration	Second administration	ICC (95% CI)

	M (SD)	M (SD)	
Chinese Facial Emotion Identification Test	14.94 (2.93)	15.53 (3.40)	0.85 (0.57–0.94)
Chinese Social Cognition Screening Questionnaire (C-SCSQ)			
Theory of mind	6.71 (2.02)	6.06 (1.56)	0.76 (0.36–0.93)
Jumping-to-conclusions	1.95 (0.92)	2.27 (0.78)	0.80 (0.43–0.94)
Paranoid/hostile attributional style	1.47 (1.12)	1.76 (1.03)	0.85 (0.60–0.95)
Neurocognitive subscale	13.41 (3.79)	12.82 (3.23)	0.67 (0.09–0.88)

### Concurrent Validity

The concurrent validity of the SCSQ was investigated by examining its relationship with clinical symptoms (BPRS), neurocognitive measures (TMT, HVLT, VF Test), and a functional measure (CWPP) (Table 3). We noted several patterns of these correlations. First, none of the correlations between clinical symptoms (BPRS) and social cognitive measures was significant. Second, all three neurocognitive test measures (TMT, HVLT, VF Test) had significant moderate correlations with the NC subscale of the C-SCSQ (absolute values of *r* ranging from 0.32 to 0.48, *p* < 0.01 to <0.05). VF was significantly correlated with all subtests of the two social cognitive tasks (absolute values of *r* ranging from 0.29 to 0.34, *p* < 0.01 to <0.05), except JTC. HVLT, a test of verbal memory, only correlated with the NC subscale of the C-SCSQ (*r* = 0.48, *p* < 0.01). TMT correlated significantly with C-FEIT, ToM, and NC (absolute values of *r* ranging from 0.25 to 0.45, *p* < 0.01 to <0.05). Third, there were only a few significant correlations between the two social cognitive tasks and functional measure (indicated by CWPP), as we examined the partial correlations controlling for age and years of education. Among the social cognitive subscales, only the PAS scale of the C-SCSQ had significant negative correlations with three out of five CWPP subscales (*r* = −0.40 to −0.46, *p* < 0.05). The other three C-SCSQ subscales (ToM, JTC, NC) had low to moderate and positive correlations with most CWPP subscales, but the correlations were not significant (probably due to the small sample size). FEIT had very low correlations with the CWPP subscales. Apart from concurrent validity, the inter-correlations between FEIT, ToM and PAS subscales of SCSQ and neurocognitive measures were in expected directions. FEIT had low correlations with ToM (*r* = 0.28), PAS (*r* = −0.27) subscales of C-SCSQ and low to medium correlations with the three neurocognitive test measures (absolute values ranging from *r* = 0.37 to *r* = 0.49). The strength of these correlations were largely comparable to previous studies which employed other social cognitive measures (2, 50).

**Table 3:** Relationship between social cognitive performance (C-FEIT and C-SCSQ) and demographic characteristics, neurocognition, and clinical symptoms.

Variables	C-SCSQ subscales				
	C-FEIT	ToM	JTC	PAS	NC
**Demographic (***n*** = 62)**					
Age	−0.32*	0.04	−1.8	−0.04	−0.36**
Gender[Table-fn tfn3]	0.04	0.16	0.10	−0.15	0.24
Years of education	0.47**	0.14	0.19	−0.05	0.42**
Illness duration	−0.15	0.17	−0.15	−0.20	−0.14

**Neurocognition (***n*** = 62)**					
Trail Making Test A^a^	−0.45**	−0.25*	−0.07	0.22	−0.40**
Hopkins Verbal Learning Test^b^	0.22	0.10	0.02	−0.12	0.48**
Verbal Fluency^b^	0.29*	0.33**	−0.04	−0.34**	0.32*

**Clinical symptoms (***n*** = 62)**					
BPRS total^c^	−0.10	−0.13	−0.18	0.09	−0.03
BPRS paranoia scale^c^	−0.07	−0.11	0.08	0.05	−0.01

**Functional measure (Chinese Work Personality Profile; ***n*** = 30)^d^**					
Task orientation	−0.11	0.38	0.22	−0.46*	0.22
Social skills	−0.00	0.22	0.28	−0.28	0.14
Self-control	0.11	0.33	0.35	−0.43*	0.10
Attitude toward supervision	−0.02	0.33	0.10	−0.40*	0.13
Personal presentation	0.20	0.29	−0.13	−0.38	0.09

*p < 0.05.

**p < 0.01.

*^a^Higher scores indicate worse performance*.

*^b^Higher scores indicate better performance*.

*^c^Higher scores indicate more clinical symptoms*.

*^d^Partial correlations are presented, adjusting for effects of age, years of education*.

*C-FEIT, Chinese Facial Identification Test; ToM, Theory of mind; JTC, Jumping-to-conclusions; PAS, Paranoid/hostile attributional style; NC, Neurocognitive subscale of SCSQ*.

### Known-Groups Validity

To compare the social cognitive performance between the patient and non-psychiatric control groups, a matched sample of patient and control groups was recruited (19 pairs). We found that there were significant differences in social cognitive performance between persons with schizophrenia and non-psychiatric controls for the C-SCSQ scores, but not for the C-FEIT scores (Table 4). As with our expectation, the non-psychiatric controls had significantly higher scores for ToM (*t* = −3.89, *p* < 0.001, *d* = 1.26), NC (*t* = −5.61, *p* < 0.001, *d* = 1.82), and lower PAS (*t* = 10.07, *p* < 0.001, *d* = −3.27) scores than the patient group. It was noteworthy that the patient group had much higher PAS scores than the controls. An unexpected finding was that the non-psychiatric control group had higher JTC tendency scores than the patient group. As the non-psychiatric control group had significantly more years of education than the patient group (*t* = −5.47, *p* < 0.0001), we conducted analysis to control this potential confounding variable. When education was added as covariate, significant results maintained that the non-psychiatric controls had significant higher scores for ToM (*F* = 13.12, *p* = 0.001), NC (*F* = 11.04. *p* = 0.002), and lower PAS (*F* = 59.13, *p* < 0.0001). We included the *t*-test result in the final table as education did not correlate with performance in the five social cognitive domains.

**Table 4:** Comparison of social cognitive performance between matched pairs of persons with schizophrenia and non-psychiatric controls.

	Persons with schizophrenia (*n* = 19)		Non-psychiatric controls (*n* = 19)				
				
	M	SD	M	SD	*t*	*p*	*d*
EP	13.63	3.9	15.47	2.5	−1.72	0.094	0.56
ToM	6.32	1.2	7.84	1.2	−3.89	<0.001	1.26
JTC	1.68	1.2	2.42	1.0	−2.05	0.047	0.67
PAS	2.93	0.6	0.68	0.7	10.07	<0.001	−3.27
NC	13.05	2.1	16.68	1.9	−5.61	<0.001	1.82

*EP, emotion perception, ToM, theory of mind, JTC, jumping-to-conclusions, PAS, paranoid/hostile attributional style, NC, neurocognitive subscale of SCSQ*.

## Discussion

The study results show that there are strengths and limitations in using C-FEIT and C-SCSQ as a set of social cognitive assessment for assessing patients with schizophrenia. First, the assessment set has good content validity and cultural relevance. After some minor modifications to the test items of the C-SCSQ, the assessment set is ready for further testing. Second, the two social cognitive tasks have fair to good test–retest reliability. FEIT and two SCSQ subscales (JTC, PAS) have ICC over 0.80, and the ToM and NC subscales of the SCSQ have fair reliability—0.76 and 0.67, respectively. Third, the neurocognitive (NC) subscale of the SCSQ correlates significantly with three standardized neurocognitive tests (TMT, HVLT, VF Test). This result supports the concurrent validity of the NC subscale. The strength and direction of correlations between FEIT, ToM, PAS, and neurocognitive tests were largely comparable to that of previous studies which employed other social cognitive assessment tasks (2, 50). Fourth, the four SCSQ subscales are able to differentiate persons with schizophrenia and non-psychiatric controls, although the FEIT scores are marginally insignificant between the two groups. In particular, the non-psychiatric controls have significantly higher ToM, and NC subscales and lower PAS scores than the persons with schizophrenia. The effect sizes are large for the ToM, PAS, and NC subscales, which support the known-groups validity of the SCSQ. However, the difference in FEIT score is small (*d* = 0.56) between the two groups. This effect size is a bit lower in magnitude than in previous studies (30). In general, these results provide support for the known-groups validity of the assessment set.

On the other hand, there are also several aspects of the results that are not completely consistent with our expectations or with the results of previous studies. First, we note that the two social cognitive assessment tasks (C-FEIT and C-SCSQ) have low and insignificant correlations with clinical symptoms (BPRS) in schizophrenia. This is not completely in line with the results of previous studies (51). This result may have arisen because of the complex interaction among psychiatric symptoms, neurocognition, and social cognition (1). Another possible explanation for this result is that the patient group had relatively few psychiatric symptoms.

Second, the ToM, PAS, and JTC subscales of the SCSQ have low-to-medium correlations with measures of functional performance (CWPP) after controlling for age and education. These results echo previous findings on the predictive significance of social cognition in functional outcomes (4, 6, 52). However, some of these correlations appear to be insignificant because of the small sample size.

Third, unlike the internal consistency of C-FEIT being acceptable (53), the internal consistency of C-SCSQ total score falls into questionable range (53). This is likely related to the inclusion of different C-SCSQ subscales that measure different but related social cognitive domains. It would be best to examine the internal consistency of C-SCSQ subscales individually. This was not conducted as some of the subscales had very limited number of items, ToM (10 items) and AS (5 items), which could significantly impact on the internal consistency (54).

There are also some limitations in the methodology that may affect the interpretation and generalizability of the study results. First, there are very few Chinese-language versions of social cognitive assessment tools available for studying the concurrent validity of the assessment set. For instance, the Faux Pas tasks and Eyes Tasks have been commonly used to measure ToM in previous studies, but these may not be the best concurrent measures as these tests measure other facets of ToM ability but not the intention-inferencing ability of the C-SCSQ. Second, more comprehensive functional measures based on performance assessment could be adopted in future studies. The CWPP is an observational assessment of patients’ functional performance in simulated work situations. One option to further improve the validity of functional assessment is to adopt performance-based tests; this is now generally preferred for more direct and more valid estimates of functional disability (55). Third, the small sample size of the study could lead to some of the unexpected findings, such as non-psychiatric controls had higher JTC score than patient group. While the effect sizes for between-group differences in other social cognitive domains could be assumed at 0.90 from previous studies, the combined effect size for JTC is 0.60 as confirmed in a recent meta-analysis (56). The finding of higher JTC score in non-psychiatric control is possibly related to the inadequate sample size. Fourth, our study used static faces in C-FEIT to measure EP ability, but some studies recommended the use of dynamic faces over static faces for better ecological validity (57, 58). These findings, however, do not have strong support from behavioral study (59). Nevertheless, there is a need to further discuss if dynamic or static faces are more valid for future studies of EP in future. Fourth, we acknowledged that an optimal test–retest interval would be 2–4 weeks (60). The use of retest interval of 1 week as it would greatly increase the probability that participants would come for retest. The sample size for evaluating test–retest reliability also needs to around 60 in order to obtain a powerful estimate of the ICC for this test (31). Last, the C-FEIT and C-SCSQ cover most aspects of social cognitive assessment, but social perception (or social knowledge) is not covered by these two tests. There is a need to include a sub-test of social perception to form a battery that provides a comprehensive evaluation of social cognition.

In summary, the C-FEIT and C-SCSQ generally possess acceptable to good psychometric properties for assessing the key social cognitive domains, including EP, intention-inferencing ability (ToM), and paranoid attributional style in persons with schizophrenia. The assessment set demonstrates satisfactory test–retest reliability and known-groups validity. Subscales of the instruments have low to moderate relationships with neurocognitive function and are predictive of some domains of functional performance. The social cognitive assessment set, consisting of the C-FEIT and the C-SCSQ, addresses the need for social cognitive assessment with a Chinese population. Nevertheless, applications of the two instruments should be carried out with caution in certain populations. The C-FEIT may not differentiate EM performance between patients with few psychotic symptoms and non-psychiatric controls, and the JTC scores of non-psychiatric controls need to be interpreted carefully. Future studies can further investigate the psychometric properties of the two assessment tasks through collecting data on discriminant validity using larger healthy and patient samples and exploring the predictive validity of the assessment set with a wider range of performance-based functional measures.

## Ethics Statement

This study was carried out in accordance with the recommendations of Hospital Authority Guide on Research Ethics with written informed consent from all subjects. All subjects gave written informed consent in accordance with the Declaration of Helsinki. The protocol was approved by the Clinical & Research Ethics Committee of the New Territories West Cluster, Hospital Authority.

## Author Contributions

PL initiates the research, conducts data collection and analysis, and drafts the manuscript. AS designs on research methodology and writes the manuscript. Both authors approve the final version to be published and agree to be accountable for all aspects of the work in ensuring that questions related to the accuracy or integrity of any part of the work are appropriately investigated and resolved.

## Conflict of Interest Statement

The authors declare that the research was conducted in the absence of any commercial or financial relationships that could be construed as a potential conflict of interest.
